# Follicle-stimulating hormone promotes age-related endometrial atrophy through cross-talk with transforming growth factor beta signal transduction pathway

**DOI:** 10.1111/acel.12278

**Published:** 2014-11-13

**Authors:** Dan Zhang, Jingyi Li, Gufeng Xu, Runjv Zhang, Chengliang Zhou, Yeqing Qian, Yifeng Liu, Luting Chen, Bo Zhu, Xiaoqun Ye, Fan Qu, Xinmei Liu, Shuai Shi, Weijun Yang, Jianzhong Sheng, Hefeng Huang

**Affiliations:** 1 Key Laboratory of Reproductive Genetics, Zhejiang University, Ministry of EducationHangzhou, 310006, China; 2 Department of Reproductive Endocrinology, Women's Hospital, School of Medicine, Zhejiang UniversityHangzhou, Zhejiang, 310006, China; 3 Department of Clinical Laboratory, Women's Hospital, School of Medicine, Zhejiang UniversityHangzhou, Zhejiang, 310006, China; 4 Institute of Cell Biology and Genetics, College of Life Sciences, Zhejiang UniversityHangzhou, Zhejiang, 310058, China; 5 Department of Pathophysiology, School of Medicine, Zhejiang UniversityHangzhou, Zhejiang, 310000, China; 6 International Peace Maternity and Child Health Hospital, Shanghai Jiao Tong University School of MedicineShanghai, 200030, China

**Keywords:** aging, atrophy, autophagy, follicle-stimulating hormone, menopause, transforming growth factor beta

## Abstract

It is widely believed that endometrial atrophy in postmenopausal women is due to an age-related reduction in estrogen level. But the role of high circulating follicle-stimulating hormone (FSH) in postmenopausal syndrome is not clear. Here, we explored the role of high circulating FSH in physiological endometrial atrophy. We found that FSH exacerbated post-OVX endometrial atrophy in mice, and this effect was ameliorated by lowering FSH with Gonadotrophin-releasing hormone agonist (GnRHa). *In vitro*, FSH inhibited endometrial proliferation and promoted the apoptosis of primary cultured endometrial cells in a dose-dependent manner. In addition, upregulation of caspase3, caspase8, caspase9, autophagy-related proteins (ATG3, ATG5, ATG7, ATG12 and LC3) and downregulation of c-Jun were also observed in endometrial adenocytes. Furthermore, smad2 and smad3 showed a time-dependent activation in endometrial cells which can be partly inhibited by blocking the transforming growth factor beta receptor II (TβRII). In conclusion, FSH regulated endometrial atrophy by affecting the proliferation, autophagy and apoptosis of endometrial cells partly through activation of the transforming growth factor beta (TGFβ) pathway.

## Results and discussion

The role of follicle-stimulating hormone (FSH) in endometrial atrophy has not been clearly defined. A significant finding that FSH directly increased osteoclastogenesis and resorption in postmenopausal women (Sun *et al*., 2006) challenged the traditional viewpoint that osteoporosis of postmenopausal women is solely related to declining estrogen levels. Later, researchers indicated the uterus of FSH receptors (FSHR) knocked out mice weighted twofolds more than that of wild-type mice (Danilovich *et al*., 2002; Abel *et al*., 2003). We hypothesized that high circulating FSH also plays a key role in endometrial atrophy in postmenopausal women.

**Figure 1:**
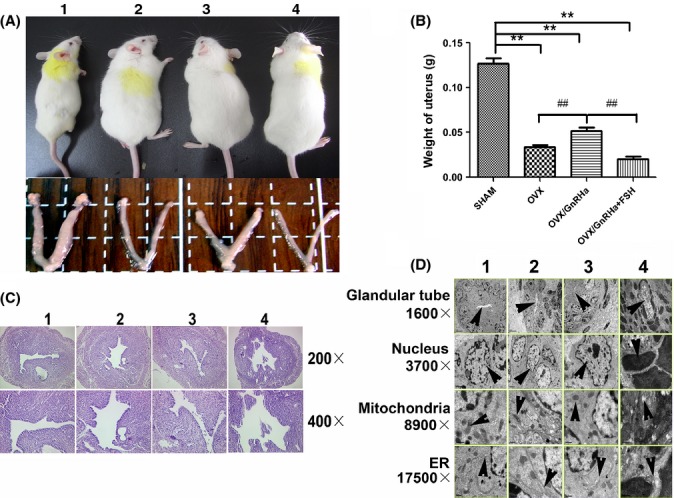
The appearance of animal models and the weight and morphological features of their uterus: OVX led to larger size of animal model (A2), but decreased size (A2) and weight of uterus in appearance (B), decreased thickness of endometrium in microscope (C2), and a series of ultrastructural changes including smaller glandular tube, more pyknotic nuclei, vacuolization in mitochondria, and hollowed rough endoplasmic reticulum (D2). The above changes were partly reversed by GnRHa treatment (A3, B, C3, D3), but were aggravated by follicle-stimulating hormone (FSH) administered together with the GnRHa (A4, B, C4, D4) (1.SHAM (low FSH, low LH, high E2); 2.OVX (high FSH, high LH, low E2); 3. OVX/GnRHa (low FSH, low LH, low E2) 4.OVX/GnRHa + FSH (high FSH, low LH, low E2); **Significant difference *P* < 0.01 vs. SHAM; ^##^Significant difference *P* < 0.01 vs. OVX/GnRHa, *n* = 6 for each group).

We validated the expression of FSHR on human uterus and endometrial cells (Fig. S2, Supporting information). We built a high FSH mice model by performing ovariectomy (OVX) (Fig.1A). Elevation of serum FSH and reduction of serum estradiol (E2) (Table S1, Fig. S1, Supporting information) accompanied by significant decrease in uterine size and weight (Fig.1B) after surgery were confirmed. The OVX-induced decreased uterine size and weight was suppressed by administration of gonadotropin-releasing hormone agonist (GnRHa) (0.5 μg day^−1^) (Fig.1A,B), which inhibited the release of FSH and LH from the pituitary (Hsueh & Erickson, 1979). Administration of recombinant FSH (0.15 IU day^−1^) in GnRHa (0.5 μg day^−1^) pretreated OVX animal model led to decreases in uterine size and weight (Fig1A,B), which may be attributed to the elevation of FSH level. Taken together, these results demonstrated that high level of FSH, but not LH, may contribute to the atrophy of the uterus in animals after OVX, a condition mimicking the postmenopausal period.

Morphological analysis showed significant decrease of endometrial thickness after OVX, which was partly rescued by GnRHa administration and aggravated by additional FSH administration (Fig.1C). The ultrastructure of the endometrial cell from OVX mice showed changes involved with apoptosis. Those changes were partly reversed by GnRHa administration, but were aggravated by additional FSH administered together with the GnRHa (Fig.1D). Those changes included the smaller size of glandular tube, pyknotic nuclei, vacuolization in mitochondria and hollowed rough endoplasmic reticulum.

**Figure 2:**
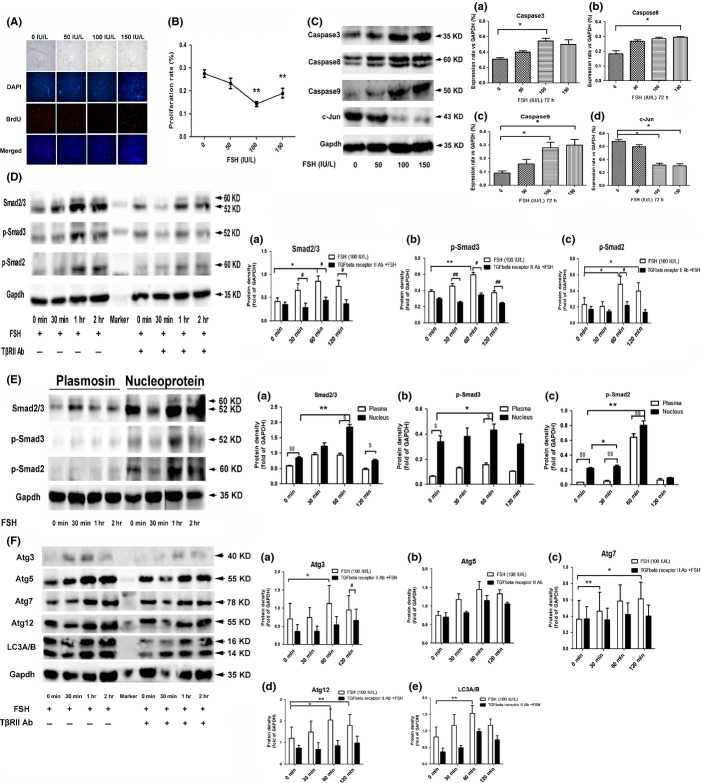
The effects of follicle-stimulating hormone (FSH) on the proliferation of primary cultured endometrial adenocytes and involvement of TGFβ pathway. FSH inhibited the proliferation of endometrial adenocytes in a dose-dependent way (A, B). The expressions of caspase3/8/9 were upregulated, and the expression of c-Jun was downregulated by FSH(C). FSH promoted the activation and nucleus translocation of p-Smad2 and p-Smad3 in a time-dependent way, which was partly recovered by antibody against TβRII (D, E). FSH upregulated ATG3, ATG5, ATG12, ATG7, and LC3A/B in a time-dependent way, which was partly recovered by antibody against TβRII (F) (*Significant difference *P* < 0.05 vs. 0 min or 0 IU L^−1^ group; **Significant difference *P* < 0.01 vs. 0 min or 0 IU L^−1^ group; ^#^Significant difference *P* < 0.05 vs. FSH group; ^##^Significant difference *P* < 0.01 vs. FSH group; ^$^Significant difference *P* < 0.05 vs. plasma group; ^$$^Significant difference *P* < 0.01 vs. plasma group, *n* = 3).

*In vitro*, we found that FSH dose-dependently inhibited the proliferation of endometrial adenocytes by 5-Bromo-2-deoxyUridine (BrdU) assay, the maximal inhibition concentration was 100 IU L^−1^ (Fig.2A,B). Furthermore, FSH upregulated cell apoptosis markers (caspase3, caspase8 and caspase9) (Fig.2C a/b/c) and downregulated proliferation-related gene c-Jun (Fig.2C d). These findings suggest that FSH plays a role in promoting endometrial atrophy directly.

Autophagy is a process of self-degradation that maintains cellular viability during periods of metabolic stress such as aging (van der Vaart *et al*., 2008). However, if the stress state persisted, apoptosis occurred (Levine & Yuan, 2005). Autophagy involves several proteins which are known as autophagy-related or ATG proteins, such as ATG3, ATG5, ATG7, ATG12, BECLINE, and LC3 (Pankiv *et al*., 2007). Our results showed FSH upregulated the protein level of ATG3, ATG7, ATG5, ATG12, and LC3A/B in a time-dependent manner, which reached a plateau at 1 h after FSH treatment (Fig.2F). We also found that the endometrium of young women undergoing controlled ovarian stimulating (COS) treatment with a high level of both FSH and E2 displayed more apoptosis bodies and the apoptosis bodies disappeared 3 months after the cycle (data unpublished). Thus, endometrial cells exposed to high levels of FSH may go through a state of autophagy at first, and persistent exposure may eventually lead to apoptosis. If FSH inhibition therapy is given in time, the state of autophagy can be completely reversed.

Transforming growth factor beta (TGFβ) is an important regulator of the proliferation, autophagy, apoptosis, invasive properties, and migration of cells (Derynck *et al*., 2001).Xavier Gueripel *et al*. (2004) reported increased expression of the TGFβ signaling pathway molecules in a model of immature mice exposed to FSH and LH. Here, we found that FSH promoted time-dependent phosphorylation and nuclear translocation of Smad2/Smad3, and the alterations can be partly reversed by pretreatment with antibody against TGFβ receptor II (TβRII) (Fig.2D,E). Similarly, the upregulation of ATGs described above by FSH treatment can also be partly reversed by pretreatment with antibody against TβRII (Fig.2F). These data showed a cross-talk between FSH and the TGFβ signal system in regulating the proliferation and autophagy of endometrial cells, partly mediated by TβRII.

In conclusion, our research showed that high circulating levels of FSH in postmenopausal women activated the phosphorylation of Smad2/Smad3 through TβRII. The complexes of phosphorylated Smad2/Smad3 subsequently transported into the nucleus and launched the expression and activation of several autophagy-related molecules such as ATG5,ATG12,ATG3, and ATG7. Persistent activation of cell autophagy may directly or indirectly induce cell apoptosis (Fig. S3, Supporting information). Whether the effects of a high level of FSH on endometrial atrophy are synergistic with low level of estrogen or whether it is a novel, direct, and independent mechanism for endometrial atrophy in postmenopausal women is unknown. Modulation and control of circulating FSH may well offer a tool for the targeted treatment of endometrial pathology.

## Supporting Information

**Fig. S1** The levels of serum FSH and E2 in animal models.

**Fig. S2** The expression of FSHR in endometrial tissue and cells.

**Fig. S3** The possible mechanisms involved in the regulation of FSH on endometrial cells.

**Table S1** The levels of serum FSH and E2 in animal models.
