# Supplementary material for: Follicle-stimulating hormone promotes age-related endometrial atrophy through cross-talk with transforming growth factor beta signal transduction pathway
Source: Aging Cell. 2014 Nov 13;14(2):284–7. doi: 10.1111/acel.12278 (PMC4364840; doi:10.1111/acel.12278)
Supplement: Supplementary file 1 [file acel0014-0284-sd1.doc]

**
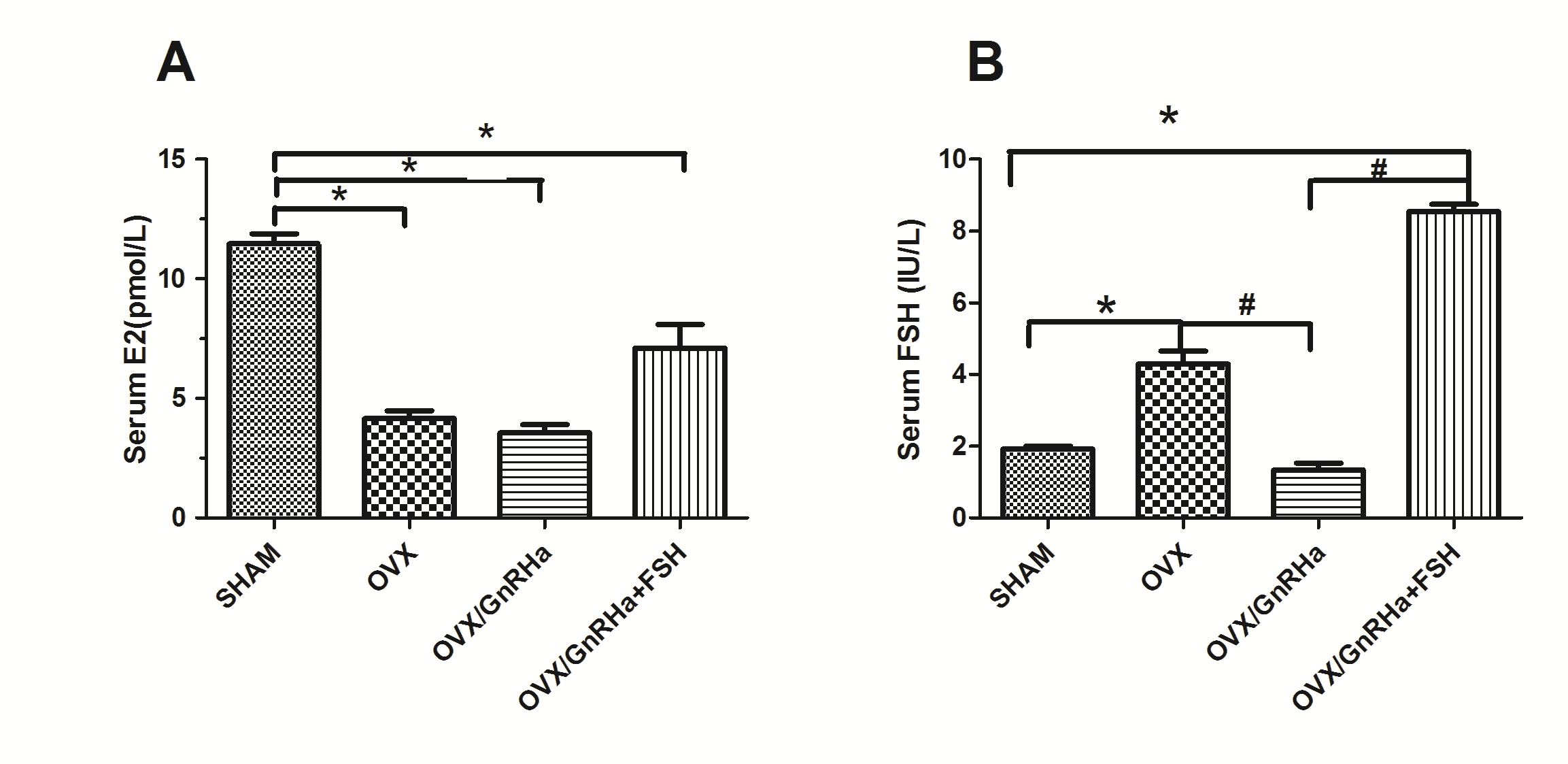
**

**Supplement Figure 1: The levels of serum FSH and E2 in animal models.** All the data were analyzed though One way ANOVA(for Comparison among groups) and Turkey’s post hoc tests (for Comparison between groups) and all the values were represent with means±SEM.(*Signiﬁcant difference (P <0.05)from wild-type values; ^#^Signiﬁcant difference (P <0.05)from OVX/GnRHa values.).
