# Supplementary material for: Follicle-stimulating hormone promotes age-related endometrial atrophy through cross-talk with transforming growth factor beta signal transduction pathway
Source: Aging Cell. 2014 Nov 13;14(2):284–7. doi: 10.1111/acel.12278 (PMC4364840; doi:10.1111/acel.12278)
Supplement: Supplementary file 2 [file acel0014-0284-sd2.doc]

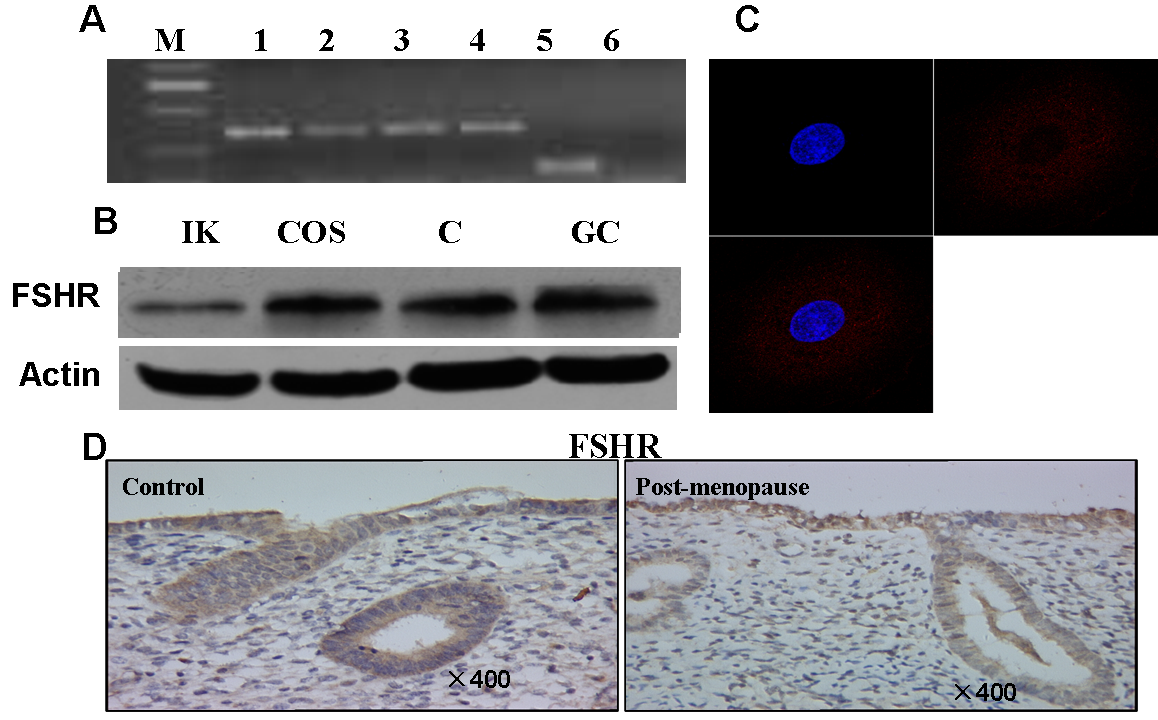


**Supplement Figure 2: the expression of FHSR in endometrial tissue and cell. A:** FSHR expression of electrophoresis M: DNA Marker, 1: Postive control(Ovary granular cell), 2:Ishikawa cells, 3&4:human endometrium, 5:negative control, 6:blank. A about 342bp band was shown on 1,2,3,4, suggesting the same mRNA have been produced. **B:** FSHR protein expression On: C: control endometrium, COS: endometrium of COS group, IK: Ishikawa cells, GC: ovary granular cells. **C:** Immunofluorescenct staining of FSHR in Ishikawa cells: Blue: DAPI stained nucleus; Dark Red: FSHR were spotted in cytoplasm and membrane. **D:** the expression of FHSR in endometrial tissue in control and postmenopausal women detected by immunohistochemistry.
