# Supplementary material for: Follicle-stimulating hormone promotes age-related endometrial atrophy through cross-talk with transforming growth factor beta signal transduction pathway
Source: Aging Cell. 2014 Nov 13;14(2):284–7. doi: 10.1111/acel.12278 (PMC4364840; doi:10.1111/acel.12278)
Supplement: Supplementary file 3 [file acel0014-0284-sd3.doc]

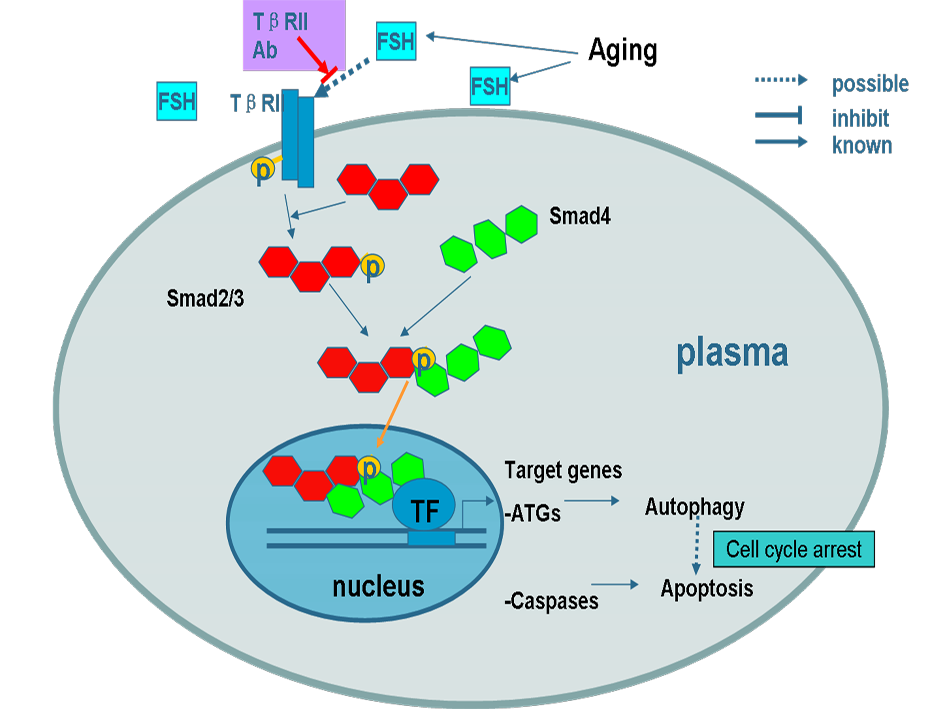


**Supplement Figure 3: The possible mechanisms involved in the regulation of FSH on endometrial cell.** High circulating FSH in post-menopausal women activated the phosphorylation of Smad2/Smad3 through TβRII. The complexus of phosphorylated Smad2/Smad3 subsequently transported into the nucleus which launch the transcription and activation of several molecules involved in the process of cell autophagy and apoptosis such as ATG5，ATG12，ATG3 and ATG7.
