# Supplementary material for: Follicle-stimulating hormone promotes age-related endometrial atrophy through cross-talk with transforming growth factor beta signal transduction pathway
Source: Aging Cell. 2014 Nov 13;14(2):284–7. doi: 10.1111/acel.12278 (PMC4364840; doi:10.1111/acel.12278)
Supplement: Supplementary file 4 [file acel0014-0284-sd4.doc]

**Table S1: The levels of serum FSH and E2 in animal models**

| **GROUPS** | **SHAM(n=6)** | **OVX(n=5)** | **OVX /GnRHa(n=5)** | **OVX/GnRHa+FSH(n=5)** | **P value** |
| --- | --- | --- | --- | --- | --- |
| **Sample** | **6** | **5** | **5** | **5** | **P<0.05** |
| **FSH (IU/L)** | **1.99±0.92** | **4.29±0.32** | **1.24±0.27** | **8.58±0.25** | **P<0.05** |
| **E2 (pmol/L)** | **11.33±0.50** | **3.74±0.21** | **3.44±0.43** | **5.61±0.32** | **P<0.05** |

**Supplement table 1: The levels of serum FSH and E2 in animal models.** All the data were analyzed though One way ANOVA(for Comparison among groups) and Tukey’s post hoc tests (for Comparison between groups) and all the values were represent with means±SEM.
