# Supplementary material for: Follicle-stimulating hormone promotes age-related endometrial atrophy through cross-talk with transforming growth factor beta signal transduction pathway
Source: Aging Cell. 2014 Nov 13;14(2):284–7. doi: 10.1111/acel.12278 (PMC4364840; doi:10.1111/acel.12278)
Supplement: Supplementary file 5 [file acel0014-0284-sd5.doc]

**Materials and Methods**

**1. Patients**

The pathological slice of uterus from 30 post menopausal women (mean age 65) who had hysterectomy for uterus/vaginal prolapsed and 30 childbearing age women (mean age 40) who had hysterectomy for leiomyoma of uterus during January in 2007 to December in 2010 in the affiliated women’s hospital of Zhejiang university were retrospectively analyzed which was approved by ethics committee of Zhejiang university affiliated women’s hospital.

**2. Animals**

8-week-old ICR female mice were castrated as ORX/OVX group to induce high endogenous gonadotropin. Gonadotrophin-releasing hormone agonist (GnRH-a, 0.5 μg per day Triptorelin; Pfizer Inc, New York, USA) was injected from the fourth day after castration for 4 weeks as ORX/OVX+GnRHa group to inhibit pituitary gonadotropins secretion. Recombinant FSH (0.15 IU per day, Merck- Serono, Geneva, Switzerland) was added for 2 weeks after castrated mice treated with GnRHa for 2 weeks as ORX/OVX+GnRHa+FSH group to obtain high exogenous FSH. The mice were housed in a 12:12 h light/dark cycle at 25 ± 0.5°C and 50-60% humidity, and were fed ad libitum with a standard diet and water.

**3. Primary cell culture**

**Endometrial sample collection and digestion**

We collect the endometrial tissue of patients undergoing uterine cavity exploration in preparation for IVF. Written informed consent was obtained from these patients. At least 1 g of epithelium (10 × 10 mm surface epithelium and 3 mm depth) was harvested from above the basement membrane of endometrium immediately following hysterectomy and under aseptic conditions. Samples were incubated with sterile DMEM/F-12 serum-free medium supplemented with penicillin (100 U/ml) and streptomycin (100 μm/ml). Tissues were washed in sterile 1* phosphate-buffered saline (PBS) at 37 °C for 20 min. Rinsed tissues were transferred to 100-mm sterile culture dishes(Corning-Costar,Cambridge, MA), diced to 5 mm^3^ pieces, then digested in 2.5 mg/ml of collagenase type IV-S( Sigma, MO, USA) at 37 °C for 2 hours with gentle agitation. Then 10ml DMEM/F12 medium with 10% FBS were added to terminate the digestion. All chemicals were purchased from Sigma-Aldrich (Saint Louis, MO, USA).

**Cells separation and adenocytes harvest**

The mixtures of digested tissues were transferred to a 50ml eppendorf tube, and then the undigested tissues were separated by filtration using an autoclaved number 100 wire cloth sieve (Newark Wire Cloth Co., Newark, NJ). Autoclaved number 400 wire cloth stainless steel micropore sieve were used to remove stroma cells. Immediately after filtration, adenocytes were collected by inverting sieves onto 100-mm culture dishes and flushing with DMEM/F12/PS. Adenocytes were enriched by centrifugation at 800 rpm at 37 °C for 5 min. Washed cells were dispersed with DMEM/F12 medium supplemented with 2 mM l-glutamine, 100 U/ml penicillin and 100 μg/ml streptomycin, and 10% (v/v) FBS, and plated into culture flasks.

**4. Western-blot**

Protein was separated by 10% (w/v) SDS-PAGE and electro-transferred onto nitrocellulose membranes (Bio-Rad Laboratories, Foster City, CA, USA), and incubated in blocking buffer (20 mM Tris, pH 7.6, 137 mM NaCl, 0.05% (v/v) Tween 20, and 1% bovium serum albumin) for 2 h followed by incubating at 4°C overnight with primary polyclonal rabbit antibodies against caspase3/8/9, c-Jun, p-smad2, p-smad3, smad2/smad3, ATG3, ATG5, ATG7, ATG12, LC3A/B( Cell Signaling Technology Boston, USA; 1:1000 ) or GAPDH (Santa Cruz Biotechnology, Santa Cruz, CA, USA; 1:400) .After three washes with blocking buffer, membranes were incubated with horseradish peroxidase-conjugated goat anti-mouse or goat anti-rabbit IgG secondary antibody (both 1:5000, Cell Signaling Technology). After additional washes, immunoreactive bands were visualized using enhanced chemiluminescence reagents (Beyotime). Densitometry was performed using Quantity One v4.4.0 (Bio-Rad Laboratories) with GAPDH as a loading control.

**5. Measurement of hormones in serum**

The levels of FSH and LH in serum of the patients and animal models were measured with chemiluminescent assays (Diagnostic Products Corporation, USA). The levels of estradiol (E2) in serum of the patients and animal models were detected with radioimmunoassay (RIA, provided by Jiuding Co, Ltd China). All the measurements were conducted according to the manufacturer’s instruction.

**6. BrdU**

Analysis of cell division was performed by BrdU assay. Primary cultured endometrial adenocytes were cultured in 6-well plates and were then incubated with complete medium supplemented with BrdU (12 mM Sigma, MO, USA) for 10h and FSH in different doses from 50IU/L to 150IU/L for another 72 hrs. After fixation with 4% paraformaldehyde/PBS for 50 min at room temperature, the cells were incubated in 2 N HCl for 30 min at 37°C and treated with blocking solution (PBS with 5% normal goat serum) for 15 min. Cells were incubated with polyclonal anti-BrdU (1:1000, Sigma, MO, USA) overnight at 4°C and incubated with secondary rhodamine-conjugated mouse monoclonal antibody (HuaAn, China) for 3–4 hrs using standard conditions. Fluorescence was viewed using a wide field fluorescence microscope. The percentages of BrdU positive cells were identified counted in the representative fields. Experiments were performed in triplicate and repeated independently at least twice for each sample. The results were reported as mean ± S.D. of three independent experiments. Comparisons between groups for statistical significance were performed with a two-tailed paired Student's t-test.

**7. TEM**

Transmission electron microscope examinations were performed to assess morphological features of endometrial adenocytes of tissues obtained from animal models of different groups according to a standard protocol. Endometrial tissue were fixed in 2.5% glutaraldehyde (in PBS) and post fixed for 1h at room temperature in 1% aqueous osmium tetroxide. Then samples were processed, and embedded in epoxyresin (Epon) according to a standard protocol. Ultrathin (1-lm-thick) sections were prepared from selected regions of epoxy-embedded implantation sites and counter stained for 10min with 4% aqueous uranylacetate, followed by 2 min of treatment with lead citrate, and viewed with a HITACH mode l7000 transmission electron microscope operated at 75kV.

**8. HE staining**

Uterine tissues were fixed in 4% paraformaldehyde in phosphate-buffered saline (PBS) for 12 hr, air-dried, and embedded in paraffin. Sections (4 µm thick) were cut, mounted on slides, deparaffinized with a graded series of xylene, and rehydrated in a descending graded alcohol series. The slides were dipped into a Coplin jar containing Mayer’s hematoxylin and agitate for 30 sec and rinsed in H_2_O for 1 min. After that the slide were then stained with 1% eosin Y solution for 10-30 sec with agitation. The sections were then dehydrated with two changes of 95% alcohol and two changes of 100% alcohol for 30 sec each. Then the alcohol was extracted with two changes of xylene. After added one or two drops of mounting medium and covered with a coverslip, slides with HE staining were finished. Image-Pro Plus (Media Cybernetics, Bethesda, MD, USA) was used to quantify images.

**9. Statistics**

All data were in normal distribution and were expressed as mean ± standard deviation (SD). The independent samples *t* test was used to evaluate the statistical significance between two groups. One-way analysis of variance (ANOVA) and LSD post hoc tests were used to evaluate the statistical significance of difference between more than two groups. We used SPSS 16.0 for Windows (SPSS, Inc.) for the statistical analysis. *P*<.05 was considered statistically significant.
